# Interactions with the young down-regulate adult olfactory neurogenesis and enhance the maturation of olfactory neuroblasts in sheep mothers

**DOI:** 10.3389/fnbeh.2014.00053

**Published:** 2014-02-18

**Authors:** Maïna Brus, Maryse Meurisse, Matthieu Keller, Frédéric Lévy

**Affiliations:** ^1^INRA, UMR 85, Physiologie de la Reproduction et des ComportementsNouzilly, France; ^2^CNRS, UMR 7247Nouzilly, France; ^3^Université François RabelaisTours, France; ^4^IFCENouzilly, France

**Keywords:** olfactory bulb, hippocampus, maternal behavior, olfactory learning, brain plasticity

## Abstract

New neurons are continuously added in the dentate gyrus (DG) and the olfactory bulb of mammalian brain. While numerous environmental factors controlling survival of newborn neurons have been extensively studied, regulation by social interactions is less documented. We addressed this question by investigating the influence of parturition and interactions with the young on neurogenesis in sheep mothers. Using Bromodeoxyuridine, a marker of cell division, in combination with markers of neuronal maturation, the percentage of neuroblasts and new mature neurons in the olfactory bulb and the DG was compared between groups of parturient ewes which could interact or not with their lamb, and virgins. In addition, a morphological analysis was performed by measuring the dendritic arbor of neuroblasts in both structures. We showed that the *postpartum* period was associated with a decrease in olfactory and hippocampal adult neurogenesis. In the olfactory bulb, the suppressive effect on neuroblasts was dependent on interactions with the young whereas in the DG the decrease in new mature neurons was associated with parturition. In addition, dendritic length and number of nodes of neuroblasts were significantly enhanced by interactions with the lamb in the olfactory bulb but not in the DG. Because interactions with the young involved learning of the olfactory signature of the lamb, we hypothesize that this learning is associated with a down-regulation in olfactory neurogenesis and an enhancement of olfactory neuroblast maturation. Our assumption is that fewer new neurons decrease cell competition in the olfactory bulb and enhance maturation of those new neurons selected to participate in the learning of the young odor.

## Introduction

In most mammals, newborn neurons are continuously provided in two main structures of the brain throughout life, the dentate gyrus (DG) of the hippocampus and the olfactory bulb (Ming and Song, 2005; Curtis et al., 2007; Brus et al., 2013). In the DG, stem cells reside in the subgranular zone (SGZ) and give rise to neuroblasts which become new granule neurons in the overlying granule cell layer (GCL; Ming and Song, 2005). In the olfactory system, neural stem cells function as primary precursors in the subventricular zone (SVZ) located on the wall of the lateral ventricles. These cells produce transient amplifying cells which rapidly divide to produce neuroblasts. The neuroblasts migrate toward the olfactory bulb along the rostral migratory stream. After reaching the olfactory bulb, the new cells migrate radially and mature into granular interneurons for the majority of them (Rochefort and Lledo, 2005).

In rodents, a growing body of literature aiming to block neurogenesis by using various methodologies shows the role of hippocampal neurogenesis in some forms of hippocampus–dependent learning tasks despite some inconsistent results (Deng et al., 2010; Arruda-Carvalho et al., 2011; Gu et al., 2012). Similarly, olfactory neurogenesis is involved in the memory processing of olfactory cues (Lazarini and Lledo, 2011). Blocking neurogenesis impaired olfactory perceptual learning (Moreno et al., 2009), short-term and long-term memory (Breton-Provencher et al., 2009; Lazarini et al., 2009; Sultan et al., 2010). In addition it has been recently demonstrated that immediate activation of newborn olfactory neurons, by using an optogenetic approach, enhances discrimination learning and memory when the task is difficult (Alonso et al., 2012). A common role in pattern separation has been proposed for newborn hippocampal and olfactory neurons and adult neurogenesis could constitute an adaptive mechanism to optimally encode contextual or olfactory information (for review, see Sahay et al., 2011).

While the implication of adult neurogenesis has been demonstrated in spatial and olfactory learning, a better understanding of the role of adult born neurons in an ethological context has begun to emerge. Social environment can modify hippocampal neurogenesis. Decrease in cell proliferation induced by social isolation rearing could be reversed by subsequent group rearing (Lu et al., 2003). Exposure to chronic social stress dramatically decreases cell proliferation in the DG of rats, mice and tree shrews (Gould et al., 1997; Czeh et al., 2002; Mitra et al., 2006). In a socio-sexual context, exposure to the male urine compounds that are involved in mate recognition increased the survival of granule cells in the accessory olfactory bulb, the main olfactory bulb (MOB), and the DG of female mice (Mak et al., 2007; Oboti et al., 2011). Moreover, suppression of neurogenesis by an anti-mitotic agent prevented mate recognition (Oboti et al., 2011) and the display of preference for dominant male in female mice (Mak et al., 2007). Another approach to assess the role of adult neurogenesis consists in evaluating whether newly-generated neurons might functionally integrate the olfactory network which process olfactory information. In male hamsters, double immunohistochemistry labeling for Fos, a marker of cell activation, and neuronal nuclei (NeuN), a marker of post-mitotic neurons, shows that olfactory bulb cells born in adulthood are activated by socio-sexual stimuli such as estrous female or aggressive male (Huang and Bittman, 2002).

A link between adult neurogenesis and parenting has not been clearly established yet (Lévy et al., 2011). Some studies indicate a regulation of neurogenesis by parturition and the onset of motherhood. In all of rodent species studied so far parturition and the early *postpartum* period are accompanied by a significant decrease in cell proliferation in the hippocampus. In primiparous mother rats, this was reported at *postpartum* day 1, 2 and 8 (Darnaudery et al., 2007; Leuner et al., 2007; Pawluski and Galea, 2007) although no effect was observed later, at *postpartum* day 28 and after weaning (Leuner et al., 2007). Parturition and early *postpartum* period do not stimulate cell proliferation in the SVZ of mice but an increase is observed at 7 days *postpartum* (Shingo et al., 2003). Surprisingly, whether cell survival in the DG or in the MOB is altered during parturition and early *postpartum* period at the onset of maternal behavior is not known in rodents. Rather, cell survival in the DG of rats was assessed either at *postpartum* day 14 (Darnaudery et al., 2007) or 21 (Pawluski and Galea, 2007) and both studies report a significant decrease when compared to virgins. A few studies have investigated the importance of stimuli provided by neonatal pups but outside the context of parturition. Nulliparous rats exposed to pups show increased cell proliferation in the DG when compared to nulliparous females regardless of their parental response (Pawluski and Galea, 2007). Likewise, virgin female prairie voles exposed to pups exhibit increased hippocampal cell proliferation (Ruscio et al., 2008). Although an increase in cell survival in the DG was reported in virgin females 21 days after pup-exposure (Pawluski and Galea, 2007), the influence on survival of the newborn neurons either in the DG or in the MOB at the time of pup-exposure is not known. The consequences of neurogenesis ablation on the onset of maternal behavior have been investigated in mice. Irradiation of the SVZ induces minor disturbances of maternal behavior (Feierstein et al., 2010b). However, infusion of an anti-mitotic agent which transiently impairs both hippocampal and olfactory neurogenesis has been shown to affect maternal behavior but only when animals are tested in an anxiogenic environment (Larsen and Grattan, 2010), whereas genetic manipulations inducing profound and long-term alterations of neurogenesis impair nursing behavior in the home cage (Sakamoto et al., 2011).

In sheep, a down-regulation of cell proliferation has been observed in mothers in contact with their lambs for 2 days both in the DG and the SVZ (Brus et al., 2010). However there has been no report examining a change in survival of newly-born neurons in the DG or the MOB that could occur during the early *postpartum* period. In addition, no study has disentangled the influence of parturition and the first interactions with the young on cell survival and this could improve our understanding of the contribution of neurogenesis to maternal behavior. In this context, maternal behavior in sheep constitutes an interesting model in which endocrine changes occurring at parturition and olfaction play a central role (Lévy et al., 1995; Lévy and Keller, 2008). In addition, not only infantile odors become very potent stimuli allowing the development of maternal care but they also provide a basis for individual recognition of the offspring. Ewes develop discriminative maternal care, called maternal selectivity, favoring their own young at suckling while rejecting any alien young. This recognition is based on the learning of olfactory characteristics of the lamb and takes place within the first hours after parturition (Lévy et al., 2004; Lévy and Keller, 2009). Some of the learning mechanisms reside in extensive neurochemical changes occurring in the MOB at parturition (Lévy et al., 1993; Lévy and Keller, 2009). In addition to these neurochemical changes, olfactory neurogenesis could provide another mechanism through which olfaction can contribute to the onset of maternal behavior and associated learning.

The aim of the present study was to evaluate the influence of parturition and learning of the lamb odor on the survival of newborn neurons. Bromodeoxyuridine (BrdU), a marker of cell division, was used in combination with two markers of neuronal maturation (doublecortin (DCX), an early maturation marker and NeuN), to compare both hippocampal and olfactory neurogenesis between virgins and parturient ewes which could interact or not with their lamb. In addition, because learning accelerates the maturation of the dendritic trees of newborn neurons in the DG (Tronel et al., 2010; Lemaire et al., 2012), and motherhood is accompanied by changes in the morphology of newborn neurons in the MOB (Kopel et al., 2012), we assessed the influence of lamb olfactory learning on this maturation by measuring the dendritic length and the number of nodes of new neuroblasts.

## Materials and methods

### Animals

Experiment was conducted on 17 Ile de France ewes, of 1.5–2 years of age, from the INRA research center in Nouzilly (Indre et Loire, France) approved by local authority (agreement number E37-175-2). Animals were permanently housed indoors, with free access to water and were fed with lucerne, maize, straw and a supplement of vitamins and minerals. Animal care and experimental treatments complied with the guidelines of the French Ministry of Agriculture for animal experimentation and European regulations on animal experimentation (86/609/EEC) and were performed in accordance with the local animal regulation (authorization No. 006352 of the French Ministry of Agriculture in accordance with EEC directive). Ewes were sacrificed by a licensed butcher in an official slaughterhouse (authorization No. A37801 E37-175-2 agreement UEPAO). All efforts were made to minimize the number of animals (5–6 animals per group).

### Bromodeoxyuridine injections and tissue preparation

Four months before sacrifice, ewes were housed in an individual pen (2 × 1 m) and received four intravenous injections of BrdU, (1 injection/day, 20 mg/Kg in 0.9% saline; Sigma-Aldrich, France), a thymidine analogue incorporated into the DNA during the S-phase of the mitotic division. Doses of BrdU and timing between injections and sacrifice were based on a previous study reporting that maturation of adult-born cells both is much longer in sheep than that of rodents and that the highest proportion of new mature neurons is found at 4 months after BrdU injections (Brus et al., 2013).

### Groups

**Figure 1:**
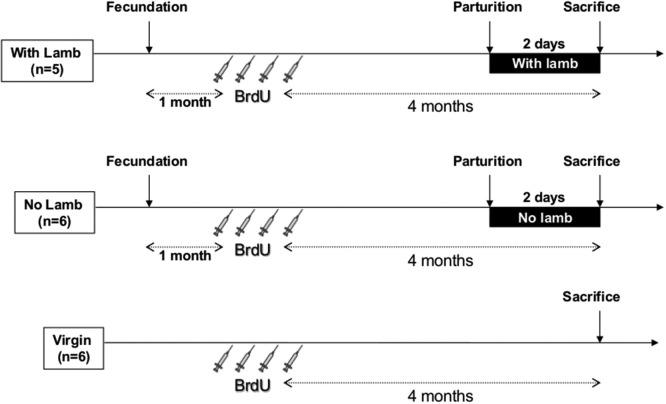
**Time table and protocol design.** All ewes received four intravenous injections of BrdU (20 mg/kg, 1 injection/day) 4 months before sacrifice. In the two parturient groups (“With Lamb” and “No Lamb”), BrdU injections were performed 1 month after fecundation. In the ”With Lamb” group (*n* = 5), ewes could interact with her lamb and maternal behavior was observed at 0, 6 and 24 h after parturition for 10 min each and selectivity test was performed at 2 days *postpartum* just before sacrifice. In the “No Lamb” group (*n* = 6), lambs were removed just after delivery. Virgin group (*n* = 6) is composed of nulliparous ewes.

Three groups of ewes were constituted (Figure 1). In parturient groups, mating was synchronized by the use of vaginal sponges containing 45 mg of fluorogestone acetate for 14 days followed by an intra-muscular injection of pregnant-mare-stimulating gonadotropins to induce ovulation. Just after parturition, mothers were either left 48 h with their lambs in their individual pen in the same barn (“With Lamb” group, *n* = 5), or were separated from them for 48 h (“No Lamb” group, *n* = 6). After being separated from their lamb immediately after birth, ewes of the “No Lamb” group were placed in a different barn to avoid any contact with lambs and ewes were housed together in a large pen to avoid stress induced by separation from the young. All the ewes had never given birth before the study. Lambing occurred within a period of gestation of 149 ± 4 days. Against all expectations, at birth the lambs displayed low vigor preventing them from feeding normally, probably due to BrdU injections in early pregnancy. Thus, in the “With lamb” group, adoptions have been performed with newborn lambs provided by the flock of the research center. It has been well established in previous studies that adoption, when performed at birth, are without any consequences on the quality of the mother-young relationship in comparison to normal mother-young lambing (Keverne et al., 1983; Kendrick et al., 1991; Lévy et al., 2010). In this group, maternal behavior was observed for 10 min at 0, 6 and 24 h after parturition to completely ensure that maternal care was normally provided to lambs. At 2 days *postpartum* just before sacrifice, selectivity was tested by presenting an alien lamb to the mother and rejection and acceptance behaviors were recorded for 3 min. The alien lamb was then taken away and the ewe was observed with her own lamb for an additional 3 min (Keller et al., 2004, 2005). These tests indicated that all the ewes of the “With Lamb” group were maternal and selective. The “Virgin” group (*n* = 6) was composed of nulliparous anoestrus ewes of similar age than the two parturient groups and housed together.

### Brain perfusion and immunohistochemistry

Two days after lambing, ewes were anesthetized with thiopental and decapitated by a licensed butcher in an official slaughterhouse. Brains were immediately perfused via carotid arteries with 2 L of 1% sodium nitrite in phosphate buffer saline, followed by 4 L of ice-cold 4% paraformaldehyde solution in 0.1M phosphate buffer (pH 7.4). The brain was dissected out, cut into blocks and post-fixed in the same fixative for 48 h. The tissues were then stored in 30% sucrose for 2 days until sectioned. Frontal sections were cut at a thickness of 30 µm using microtome or cryostat and stored at −20°C in cryoprotectant.

To reveal BrdU positive cells in the MOB and the DG, a peroxydase single-immunolabeling was used as described previously (Brus et al., 2013). To characterize the populations of BrdU positive cells which could be affected by parturition and interaction with the young, double immunolabeling was performed against the BrdU and two markers of neuronal maturation, NeuN for mature neurons (Valley et al., 2009) and the DCX for neuroblasts (Gleeson et al., 1999; Brown et al., 2003). Sections of the MOB and the hippocampus were treated with a solution of Tris Buffer Saline 0.025M (TBS, pH=7.4)-Triton 0.3%-Azide 0.1%-Bovine Serum Albumin (BSA) 0.1% (TBSTA-BSA) for 1 h. After one rinse in TBS, sections were treated with 2N HCl in TBS for 30 min at room temperature. After three rinses in TBS, sections were incubated overnight in primary rat anti-BrdU (1:300; AbCys AbC117-7513, Paris, France) and primary mouse anti-NeuN (1:1000, Chemicon MAB377, Millipore, St. Quentin-en-Yvelines, France) or primary goat anti-DCX (1:100, Santa Cruz Biotechnology, Tebu-Bio, Le Perray en Yvelines, France) in TBSTA-BSA. The following day, sections were rinsed three times in TBS, and were incubated in two secondary antibodies simultaneously for 1 h 30 min in TBS 0.025M, pH7.4—Saponine 0.3%—BSA 0.1% (TBS-Saponine-BSA), except for BrdU/DCX for which secondary antibodies were incubated in TBS-rabbit serum 1%-saponine 0.3%. Secondary antibodies used for BrdU/NeuN immunolabeling were a donkey anti-rat CY3 (1:300, Immunotech, Jackson ImmunoResearch, United Kingdom), and a goat anti-mouse 488 (1:300, AlexaFluor A11029, Molecular Probes, Eugene, Oregon, USA); for BrdU/DCX, we used a rabbit anti-rat 488 (1:300, AlexaFluor, Molecular Probes, Eugene, Oregon, USA) and a Donkey anti-goat CY3 (1:300, Immunotech, Jackson ImmunoResearch, UK). After four rinses in TBS, sections were immersed in a Hoechst bath for 2 min (Hoechst 33258, 2 µg/ml in water, Invitrogen, USA), rinsed in two baths of water and one bath of TBS (5 min each), then cover-slipped under fluoromount-G (SouthernBiotech, Birmingham, AL, USA) and stored at 4°C in dark.

Because in a previous study we observed cell proliferation within the MOB (Brus et al., 2010), we evaluated the influence of parturition and interactions with the young on cell proliferation in this olfactory structure as well as in the DG. To this end, single-immunolabeling was performed against the Ki67 marker, an endogenous marker of cell division which is expressed at all the phases of the cellular cycle (Kee et al., 2007). Sections were treated with TBSTA-BSA for 1 h and were incubated overnight in primary antibody rabbit anti-Ki67 (1:500, Abcam ab15580-25, Cambridge, UK) in TBSTA-BSA at room temperature. The following day, sections were rinsed four times in TBS and were incubated in secondary antibody sheep anti-rabbit (1:400, produced by the INRA center of Nouzilly) in TBS-BSA 0.1% during 3 h at 4°C. After four rinses in TBS 0.1%, sections were incubated with rabbit peroxydase anti peroxydase (PAP, 1:80000, Dako Z0113, Trappes, France) overnight at 4°C. The last day, after two rinses in TBS and two rinses in Tris-HCl (0.05M, pH 7.6) sections were reacted for peroxydase detection in a solution of 3,3′-Diaminobenzidine tetrahydrochloride (DAB, 0.15 mg/mL; Sigma) containing 0.001% H_2_O_2_ and 0.018% nickel ammonium sulfate for 7 min.

### Quantification

The number of BrdU+ (10 sections/animal, 800 µm between sections) and Ki67+ cells (6 sections/animal, 950 µm between sections) was assessed by counting peroxydase/DAB-stained frontal sections of the MOB and the DG through different levels along the rostrocaudal axis, using a light microscope (Axioskope 2, Zeiss, Germany) on a magnification of x20 and cell count analysis software (computerized image analysis Mercator, Explora Nova, La Rochelle, France). The counter was blind to the experimental group. Areas of the MOB (granular and periventricular layers) and the DG (GCL and SGZ) were measured with this system through an x2.5 objective (MOB) and an x10 objective (DG). Cell densities were then calculated by dividing the numbers of BrdU+ or Ki67+ cells by the layer area. The Ki67+ cell density corresponds to the mean of cell density measured in the granular and the periventricular layers for the MOB and in the GCL and the SGZ for the DG. Densities of BrdU+ cells and proportions of new neurons and neuroblasts were counted only in the target structures of newly-born cells integration, the granular layer of the MOB and the GCL of the DG.

To determine the percentage of BrdU+/NeuN+ cells and BrdU+/DCX+ cells in the MOB and in the DG, approximately 100 cells per ewe were observed for the three groups (around 500–600 cells per group). Each BrdU+ cell was analyzed in its entire *z*-axis, with 0.5 µm step intervals, through an x40 oil immersion objective, using a confocal laser-scanning microscope (LSM700, Zeiss, Germany) equipped of excitation wavelengths 488 and 555. Cells rotated in orthogonal planes to verify double labeling with NeuN or DCX. For each selected cell that showed co-localization of BrdU with NeuN or DCX, an image was collected with the software Zen (Carl Zeiss, Germany). All images shown correspond to one focal plane (0.5 µm) and were imported into Gimp Pack Mode 2.6 software to adjust brightness and contrasts.

**Figure 4:**
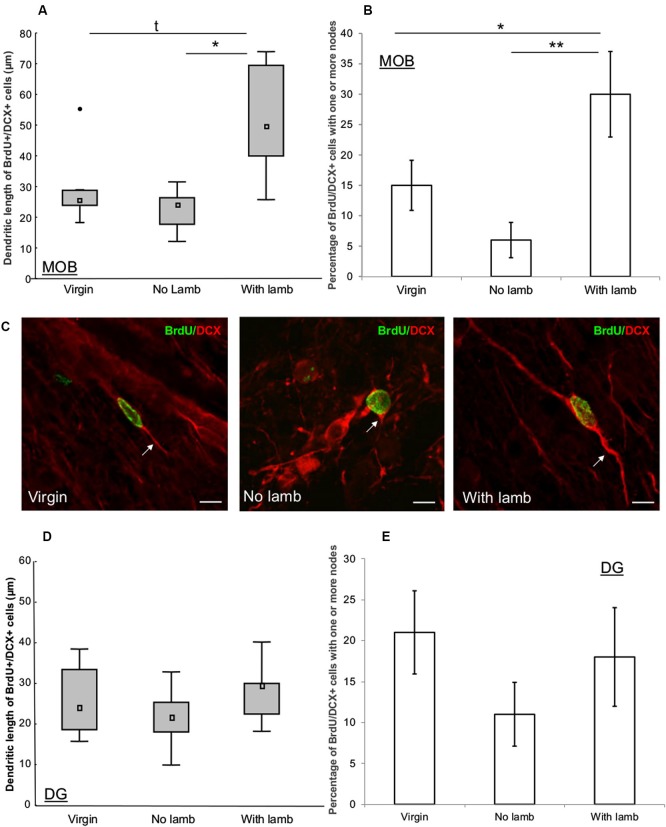
**Effects of parturition and/or interactions with the young on the dendritic tree of neuroblasts. (A, D)** Length of dendritic processes of BrdU+/DCX+ cells (16–18 cells/animal) in the granular layer of the MOB **(A)** and in the granular cell layer of the DG **(D)** in the “Virgin” (*n* = 6), the “No Lamb” (*n* = 6) and the “With Lamb” (*n* = 5) groups. **(B, E)** Percentage of BrdU+/DCX+ cells which displayed one or more nodes in each group in the granular layer of the MOB **(B)** and in the granular cell layer of the DG **(E)**. In the MOB, the “With Lamb” group showed highest length of dendritic trees and more nodes on BrdU+/DCX+ cells than in the two other groups **(A–B)**. In the DG no significant difference was found **(D–E)**. Data are represented as median and interquartile ranges. * *p* ≤ 0.05, ** *p* < 0.01. •: extreme value. **(C)** Representative illustration of dendritic lengths of DCX+/BrdU+ cells measured in the granular layer of the MOB in the “Virgin”, the “No Lamb” and the “With Lamb” groups. Scales bars: 10 µm.

To determine the development of the newborn neuroblasts in the different groups, the number of nodes and the length of the dendritic arbor were measured in 16–18 BrdU+/DCX+ cells per ewe in the GCL of the MOB and of the DG. Each BrdU+/DCX+ cell was analyzed with confocal laser scanning microscope (LSM 700, Zeiss, Germany), in its entire *Z*-axis with 0.5 µm step interval, using x63 oil immersion objective to measure the length between the cell body to the end of the longest process. Total length of the dendritic tree was obtained by summing the length of all processes of each BrdU+/DCX+ cell. The number of nodes was obtained by counting the number of occurrences of branch points in the dendritic arbor. Interestingly, these newborn cells seemed to be at an early stage of maturation as most of them displayed only one process. Thus, the population of BrdU+/DCX+ was separated in two categories depending on the number of nodes and the percentage of cells with no nodes (one process, less mature) or with one or more nodes (two or more processes, more mature) was calculated (Figures 4B–E). Ambiguous cases were further analyzed using a semiautomatic neuron tracing system Imaris (Bitplane, USA).

### Statistical analysis

As densities of BrdU+ and Ki67+ cells, proportions of BrdU+/NeuN+ and BrdU+/DCX+ cells, and dendritic lengths of BrdU+/DCX+ cells were not normally distributed, the data were analyzed with nonparametric tests (Siegel, 1956). Inter-group comparisons were analyzed using two-tailed Kruskal-Wallis and Mann-Whitney tests. As the percentage of BrdU+/DCX+ cells which displayed one or more nodes was normally distributed, the data were analyzed with a one-way analysis of variance (ANOVA), and significance was probed by the Newman-Keuls test. Statistical analyses were performed using the statistical package SPSS 10 (Chicago, IL, USA) and the level of statistical significance was set at *p* ≤ 0.05. All data were represented as median and interquartile ranges except the number of nodes which were represented as mean ± SEM. Because we found that cell proliferation is down-regulated in *postpartum* ewes (Brus et al., 2010), we predicted that density of Ki67+ cells will be lower in parturient groups and therefore we used one-tailed Kruskal-Wallis and Mann-Whitney tests for this variable.

## Results

### Survival of neuroblasts and new mature neurons in the main olfactory bulb (MOB) and the dentate gyrus (DG)

**Figure 2:**
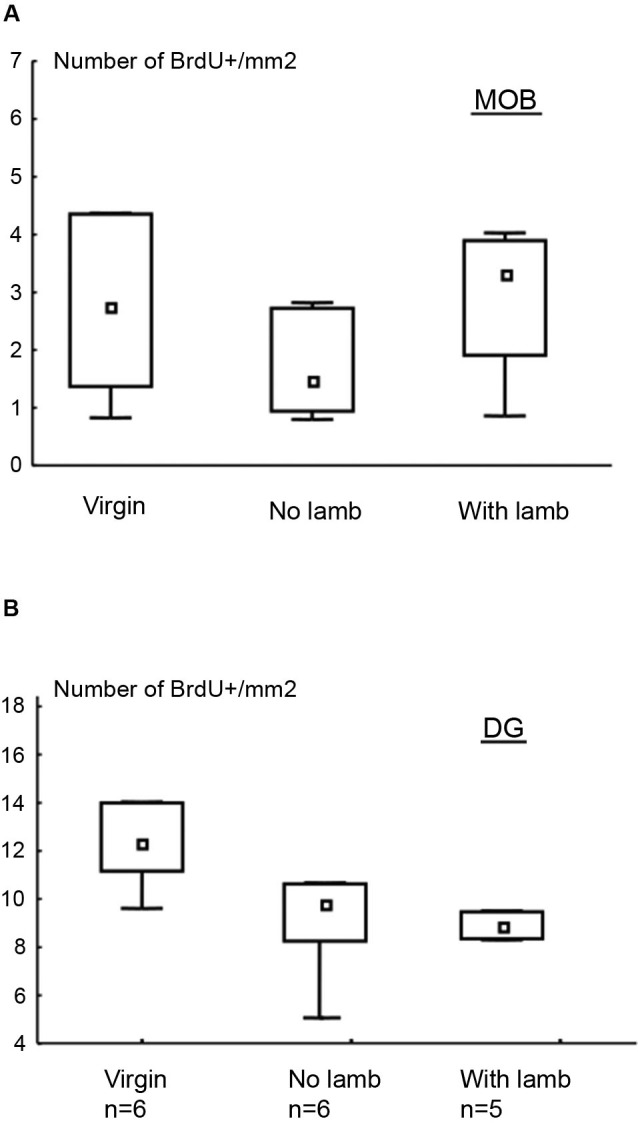
**Effects of parturition and/or interactions with the young on cell survival.** Number of BrdU+ cells per millimeter^2^ (median and interquartile ranges) in the granular layer of the MOB **(A)** and in the DG **(B)**. There is no significant difference between groups for both regions.

In the granular layer of the MOB and in the GCL of the DG, the densities of BrdU+ cells did not significantly differ between groups (MOB: *H* = 2.27, *p* = 0.3; DG: *H* = 4.48, *p* = 0.1; Figures 2A, B).

**Figure 3:**
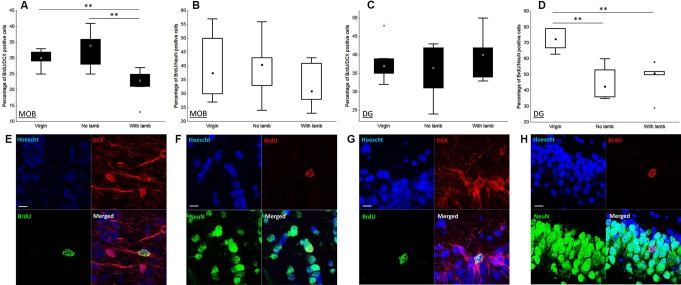
**Effects of parturition and/or interactions with the young on the proportion of neuroblasts and mature neurons.** Percentage of neuroblasts (BrdU+/DCX+) and new mature neurons (BrdU+/NeuN+) in the granular layer of the MOB **(A–B)** and in the granular cell layer of the DG **(C–D)**, in the “Virgin” (*n* = 6), the “No Lamb” (*n* = 6) and the “With Lamb” (*n* = 5) groups. Data are expressed as percentage of BrdU+ cells colabeled with NeuN **(B–D)** and DCX **(A–C)**. In the MOB, only the proportion of BrdU+/DCX+ cells in the “With Lamb” group significantly differed from the two other groups **(A)**. In the DG, only the proportion of BrdU+/NeuN+ cells in both parturient groups is significantly lower than the “Virgin” group **(D)**. **(E–H)** High magnification of confocal images of fluorescent immunolabeling depicting colocalization of BrdU+/NeuN+ **(F, H)** and BrdU+/DCX+ **(E, G)** in the granular layer of the MOB **(E–F)**, in the granular cell layer of the DG **(G–H)**. Cell nuclei are labeling with Hoechst. Scales bars: 10 µm. MOB: main olfactory bulb, DG: dentate gyrus, BrdU: bromodeoxyuridine, DCX: doublecortin, NeuN: neuronal nuclei. * *p* ≤ 0.05, ** *p* < 0.01.

The proportion of newborn neurons was measured in each group by using a double immunofluorescent labeling for BrdU and NeuN, a marker of post-mitotic neurons, and DCX, a marker of neuroblasts (Figures 3E–H). In the granular layer of the MOB, only the proportion of BrdU+/DCX+ cells significantly differed between groups (*H* = 8.27, *p* = 0.02; Figure 3A). This proportion was significantly lower in the “With Lamb” group compared to the “Virgin” or the “No Lamb” groups (“With Lamb” vs. “Virgin” or “No Lamb” groups: *U* = 2.47, *p* = 0.01; Figure 3A). The proportion of new post-mitotic neurons (BrdU/NeuN+ cells) did not differ between groups (*H* = 1.23, *p* = 0.5; Figure 3B).

Contrary to the MOB, only the proportion of BrdU+/NeuN+ cells significantly differed between groups in the GCL of the DG (*H* = 11.07, *p* = 0.004; Figure 3D). The proportion of new mature neurons was significantly lower in the two parturient groups compared to the Virgin group (“With Lamb” vs. “Virgin” groups: *U* = 0, *p* = 0.004; “No Lamb” vs. “Virgin” groups: *U* = 0, *p* = 0.002). However, the proportion of BrdU+/DCX+ cells did not significantly differ between groups (*H* = 0.73, *p* = 0.7; Figure 3C).

### Dendritic lengths of newborn neurons in the main olfactory bulb (MOB) and the dentate gyrus (DG)

To assess the influence of interactions with the young on the development of the dendritic arbor of new neurons, we measured dendritic lengths and number of nodes of BrdU+/DCX+ cells in the granular layer of the MOB and in the GCL of the DG (Figure 4).

In the MOB, dendritic lengths and number of nodes significantly differed between groups (dendritic length: *H* = 6.01, *p* = 0.049; nodes: *F* = 6.58, *p* = 0.01). The highest dendritic lengths were found in the “With Lamb” group and significantly differed from the “No Lamb” (*U* = 3, *p* = 0.03; Figure 4A) and showed a tendency to differ from the “Virgin” group due to an animal showing an extreme value (*U* = 5, *p* = 0.08; Figure 4A). The percentage of BrdU+/DCX+ cells possessing nodes was significantly greater in the “With Lamb” group than in the “Virgin” or “No Lamb” groups (“With Lamb” vs. “Virgin” groups: *p* = 0.04; “With Lamb” vs. “No Lamb” groups: *p* = 0.007; Figure 4B).

By contrast in the DG, no difference in dendritic lengths and in the number of nodes were found between groups (dendritic length: *H* = 1.35, *p* = 0.5; nodes; *F* = 1.04, *p* = 0.38; Figures 4D, E). Finally, the diameter of BrdU+/DCX+ cell bodies did not differ between groups in both structures (MOB; *F* = 2.33, *p* = 0.13; DG: *F* = 1.56, *p* = 0.24).

### Cell proliferation in the main olfactory bulb (MOB) and the dentate gyrus (DG)

**Figure 5:**
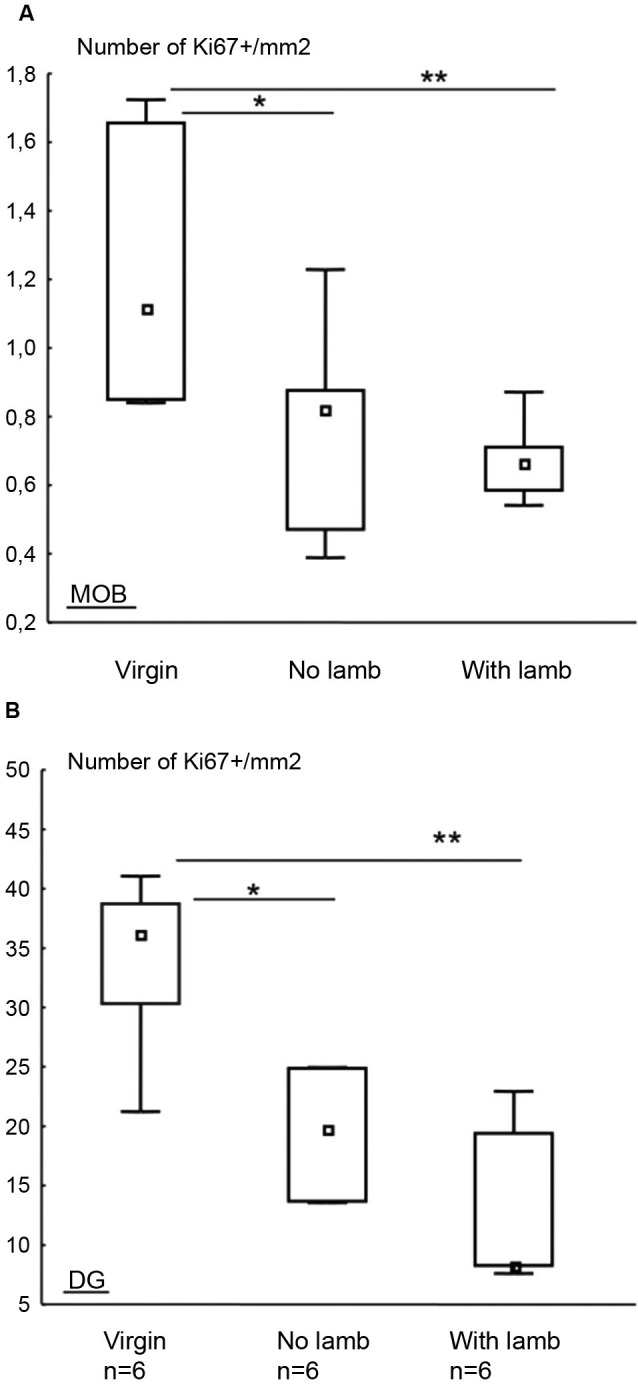
**Effects of parturition and/or interactions with the young on cell proliferation.** Densities of Ki67+ cells in the MOB **(A)** and in the DG **(B)** in the “Virgin”, “No Lamb” and “With Lamb” groups, represented as median and interquartile ranges. In both structures, the density of Ki67+ cells was significantly lower in the two parturient groups compared to “Virgin” group. * *p* ≤ 0.05, ** *p* < 0.01.

To evaluate the influence of parturition and interactions with the young on cell proliferation, a single immunolabeling was performed against the Ki67 protein, an endogen marker of cell division, in the MOB and the DG (Figure 5). In both the MOB and the DG, the density of Ki67+ cells significantly differed between groups (MOB: *H* = 6.13, *p* = 0.025, Figure 5A; DG: *H* = 7.59, *p* = 0.01, Figure 5B). For both structures, in the two parturient groups, the density of Ki67+ cells in the MOB was significantly lower compared to the “Virgin” group (MOB: “With Lamb” vs. “Virgin” groups: *U* = 2, *p* = 0.01; “No Lamb” vs. “Virgin” groups: *U* = 7, *p* = 0.04; DG “With Lamb” and the “Virgin” groups: *U* = 1, *p* = 0.01; “No Lamb” group vs. “Virgin” groups: *U* = 8, *p* = 0.05).

## Discussion

Two main results have been obtained in this study. First, interactions with the young and associated learning, but not parturition, reduce the survival of neuroblasts in the MOB, whereas in the DG, parturition induces a decrease in the number of new neurons. These results suggest that learning of the olfactory signature of the lamb, which occurred during the first mother/young interactions, is specifically associated with a down-regulation in olfactory neurogenesis. Secondly, a positive effect on the development of the dendritic length of new neuroblasts has been specifically observed in the MOB of mothers interacting with their lamb suggesting that olfactory learning accelerates the maturation of adult-born neurons in the MOB but not in the DG.

In the MOB, while the number of BrdU+ cells was similar between parturient groups and the “Virgin” group, the population of neuroblasts was decreased by interactions with the young. This reduction is probably not a consequence of a decrease in new cell production during gestation since a positive, rather than a negative, influence of pregnancy on cell proliferation has been described in the SVZ of rodents (Shingo et al., 2003; Furuta and Bridges, 2005; Larsen and Grattan, 2010). In addition, parturient ewes separated from their lambs did not show any change of cell survival in comparison to virgins indicating that parturition could not account for this down-regulation. However, the suppressive effect of interactions with the lamb for 2 days on the survival of neuroblasts could be a consequence of various behaviors involved in maternal care. For instance licking the newborn lamb depends on olfactory attraction to amniotic fluids (Lévy et al., 1983). However since licking behavior occurs at parturition and only last for 2 h it is unlikely involved in the decrease in neuroblasts population observed after 2 days of mother-young contact. Somatosensory stimulation associated with suckling could also account for this suppressive effect. However there is so far no evidence in the literature that suckling can have a direct effect on olfactory neurogenesis. In this respect, it would be of interest to assess olfactory neurogenesis in mothers whose udders are covered to prevent lambs from suckling. In rodents, restriction of suckling and tactile stimulation in lactating mothers in the presence of pups modulate neurochemical activity of the MOB, suggesting the importance of olfactory interactions with the young (Munaro, 1990). In sheep, during the early *postpartum* period various neurochemical and electrophysiological changes in the functional circuitry of the MOB underlie the formation of olfactory memory for lamb (Sanchez-Andrade et al., 2005; Lévy and Keller, 2008). Learning of the lamb odor could also be accompanied by changes in olfactory neurogenesis. Numerous studies report that bulbar neurogenesis varies following olfactory learning and that these changes could affect or not mature new neurons (see review: Feierstein et al., 2010a). However, whether these changes concern neuroblasts is not known. Here we show that neuroblasts but not more mature neurons are sensitive to social interactions. The decrease in the number of BrdU+/DCX+ cells cannot be explained by an enhancement of neuronal maturation since we did not observe an increase in BrdU+/NeuN+ cells in ewes interacting with their lambs. This decrease rather suggests that neuroblasts die during the 2 days of lamb exposure. Studies of adult-born neurons showed that olfactory learning has a complex effect on neuronal turnover, increasing or decreasing the survival of newborn olfactory cells as a function of their age (Mandairon et al., 2006; Mouret et al., 2008). Further studies using different timing of BrdU injections are needed to examine whether newborn cells of different age and maturation could have been differently affected.

Together with a reduced number of neuroblasts, a decrease in cell proliferation in the MOB is observed in both groups of parturient ewes in comparison to virgins, as previously shown (Brus et al., 2010). The functional significance of this reduced proliferation and maturation processes in the MOB remains unknown. It may accentuate a survival-promoting effect by eliminating newborn neurons that are not functional and would favor the maturation and integration of new neurons which participate in learning by reducing cell competition. Computational studies support this hypothesis by showing that the rate of cell proliferation and cell survival contribute to the stability of the neural activity in the network (Lehmann et al., 2005; Butz et al., 2006). This assumption is supported by our results showing that dendritic maturation of neuroblasts is only enhanced in mothers interacting with their lamb in the MOB.

Two main non-exclusive mechanisms could be considered to account for this accelerated maturation. Firstly, nursing and suckling could influence the morphology of new neurons as several studies report an effect of lactation on dendritic arbor in the mother brain. For instance, enhanced spine density has been reported in the hippocampus of lactating rats (Kinsley et al., 2006). Similarly lactating females have longer basal dendritic length compared to diestrous females in the medial preoptic area, a key region for maternal behavior (Keyser-Marcus et al., 2001). However, a negative effect has been reported on oxytocin neurons having less dendritic branches and a fewer total dendritic length in lactating rats compared to virgin rats (Armstrong and Stern, 1998; Stern and Armstrong, 1998). Secondly and more interestingly, learning the familiar lamb odor could induce this increased maturation as a positive influence of olfactory stimulation. The effect of odor learning on dendritic trees of newborn neurons has already been reported. Sensory deprivation drastically decreases the number, the dendritic length and the spine density of newborn granule cells in the MOB (Rochefort and Lledo, 2005) and odor enrichment induces structural synaptic plasticity of adult-born granule neurons (Livneh and Mizrahi, 2011). Since evidence of an influence of learning but not of suckling on the maturation of new adult neurons has been reported in the literature, we hypothesized that learning of the olfactory signature of the lamb is involved in this maturation process. Further studies need to be done to establish whether suckling and/or learning of the lamb odor are responsible for this increased maturation of neuroblasts and whether this could also affect new mature neurons.

In the DG, while no change in the number of BrdU+ cells was found between groups, survival of new mature neurons was reduced by parturition itself, since parturient ewes isolated from their lamb showed a decrease in BrdU+/NeuN+ cells in comparison to virgins. The effect of pregnancy on the decrease in cell production is unlikely since no changes in hippocampal neurogenesis have been reported either during early or late pregnancy in rodents (see review: Lévy et al., 2011). However we cannot exclude a species dependent regulation of neurogenesis during pregnancy. These effects could also be due to the stress induced by removal of the lamb. This is however unlikely since we observed a similar decrease in the survival of mature neurons in mothers staying with their lambs. The inhibition of neurogenesis could be the consequence of hormonal changes, mainly steroids and glucocorticoids occurring at parturition. Oestradiol induced a decrease in survival of newborn neurons (Barker and Galea, 2008) and applications of corticosterone suppress cell survival in the DG of the hippocampus (Wong and Herbert, 2004, 2006). Interestingly, oestradiol and corticosterone were found to affect neurogenesis and some interactions exist between both hormones. For example, adrenal steroids mediate the suppression of cell proliferation induced by oestradiol in ovariectomized females (Ormerod and Galea, 2001). The interplay between estradiol and cortisol at parturition may also mediate levels of cell survival in the DG and further studies are needed to better determine this complex influence on neurogenesis. In addition, our results suggest a hormonal influence depending on cell type since no change in the proportion of neuroblasts was found between groups in the DG. Recent studies report that high corticosterone differently affects population of new cells according to their maturity stage (Gonzalez-Perez et al., 2011; Lussier et al., 2013). Different effects on different cell lineages may represent adaptative actions of glucocorticoids, which provide a compensatory mechanism to protect some types of cells from death in the hippocampus. Such a mechanism would prevent from a dysfunction of the hippocampus during the *postpartum* period and would allow a better spatial memory in lactating females (Kinsley et al., 1999).

In conclusion, this study supports the hypothesis that the maternal brain undergoes neuronal changes, such as adult neurogenesis, which could constitute an adaptive response to motherhood by favoring learning ability. More specifically, interactions with the young are associated with a down-regulation in olfactory neurogenesis and an enhancement of neuroblast maturation. Our hypothesis is that fewer new neurons decrease cell competition in the olfactory bulb and enhance maturation of those new neurons selected to participate in the learning of the lamb odor. Further experiments will aim at understanding how these adult born neurons could be integrated to the neural network involved in maternal behavior.

## Author contributions

Maïna Brus, Maryse Meurisse, Matthieu Keller and Frédéric Lévy designed and performed research, analyzed data and wrote the paper.

## Conflict of interest statement

The authors declare that the research was conducted in the absence of any commercial or financial relationships that could be construed as a potential conflict of interest.

## References

[B1] Alonso M. Lepousez G. Sebastien W. Bardy C. Gabellec M. M. Torquet N. (2012). Activation of adult-born neurons facilitates learning and memory. Nat. Neurosci. 15, 897–904 10.1038/nn.3108 22581183

[B2] Armstrong W. E. Stern J. W. (1998). Phenotypic and state-dependent expression of the electrical and morphological properties of oxytocin and vasopressin neurones. Prog. Brain. Res. 119, 101–113 10.1016/s0079-6123(08)61564-2 10074783

[B3] Arruda-Carvalho M. Sakaguchi M. Akers K. G. Josselyn S. A. Frankland P. W. (2011). Posttraining ablation of adult-generated neurons degrades previously acquired memories. J. Neurosci. 31, 15113–15127 10.1523/jneurosci.3432-11.2011 22016545 PMC6623574

[B4] Barker J. M. Galea L. A. (2008). Repeated estradiol administration alters different aspects of neurogenesis and cell death in the hippocampus of female, but not male, rats. Neuroscience 152, 888–902 10.1016/j.neuroscience.2007.10.071 18353559

[B5] Breton-Provencher V. Lemasson M. Peralta M. R. 3rd. Saghatelyan A. (2009). Interneurons produced in adulthood are required for the normal functioning of the olfactory bulb network and for the execution of selected olfactory behaviors. J. Neurosci. 29, 15245–15257 10.1523/jneurosci.3606-09.2009 19955377 PMC6665973

[B6] Brown J. P. Couillard-Despres S. Cooper-Kuhn C. M. Winkler J. Aigner L. Kuhn H. G. (2003). Transient expression of doublecortin during adult neurogenesis. J. Comp. Neurol. 467, 1–10 10.1002/cne.10874 14574675

[B7] Brus M. Meurisse M. Franceschini I. Keller M. Levy F. (2010). Evidence for cell proliferation in the sheep brain and its down-regulation by parturition and interactions with the young. Horm. Behav. 58, 737–746 10.1016/j.yhbeh.2010.07.006 20692260

[B8] Brus M. Meurisse M. Gheusi G. Keller M. Lledo P. M. Levy F. (2013). Dynamics of olfactory and hippocampal neurogenesis in adult sheep. J. Comp. Neurol. 521, 169–188 10.1002/cne.23169 22700217

[B9] Butz M. Lehmann K. Dammasch I. E. Teuchert-Noodt G. (2006). A theoretical network model to analyse neurogenesis and synaptogenesis in the dentate gyrus. Neural. Netw. 19, 1490–1505 10.1016/j.neunet.2006.07.007 17014989

[B10] Curtis M. A. Kam M. Nannmark U. Anderson M. F. Axell M. Z. Wikkelso C. (2007). Human neuroblasts migrate to the olfactory bulb via a lateral ventricular extension. Science 315, 1243–1249 10.1126/science.1136281 17303719

[B11] Czeh B. Welt T. Fischer A. K. Erhardt A. Schmitt W. Muller M. B. (2002). Chronic psychosocial stress and concomitant repetitive transcranial magnetic stimulation: effects on stress hormone levels and adult hippocampal neurogenesis. Biol. Psychiatry 52, 1057–1065 10.1016/s0006-3223(02)01457-9 12460689

[B12] Darnaudery M. Perez-Martin M. Del Favero F. Gomez-Roldan C. Garcia-Segura L. M. Maccari S. (2007). Early motherhood in rats is associated with a modification of hippocampal function. Psychoneuroendocrinology 32, 803–812 10.1016/j.psyneuen.2007.05.012 17640823

[B13] Deng W. Aimone J. B. Gage F. H. (2010). New neurons and new memories: how does adult hippocampal neurogenesis affect learning and memory? Nat. Rev. Neurosci. 11, 339–350 10.1038/nrn2822 20354534 PMC2886712

[B14] Feierstein C. E. Lazarini F. O. Wagner S. Gabellec M.-M. De Chaumont F. Olivo-Marin J.-C. (2010b). Disruption of adult neurogenesis in the olfactory bulb affects social interaction but not maternal behavior. Front. Behav. Neurosci. 4:12 10.3389/fnbeh.2010.00176 21160552 PMC3001759

[B15] Feierstein C. E. Lazarini F. Wagner S. Gabellec M. M. De Chaumont F. Olivo-Marin J. C. (2010a). Disruption of adult neurogenesis in the olfactory bulb affects social interaction but not maternal behavior. Front. Behav. Neurosci. 4:176 10.3389/fnbeh.2010.00176 21160552 PMC3001759

[B16] Furuta M. Bridges R. S. (2005). Gestation-induced cell proliferation in the rat brain. Brain Res. Dev. Brain Res. 156, 61–66 10.1016/j.devbrainres.2005.01.008 15862628

[B17] Gleeson J. G. Lin P. T. Flanagan L. A. Walsh C. A. (1999). Doublecortin is a microtubule-associated protein and is expressed widely by migrating neurons. Neuron 23, 257–271 10.1016/s0896-6273(00)80778-3 10399933

[B18] Gonzalez-Perez O. Chavez-Casillas O. Jauregui-Huerta F. Lopez-Virgen V. Guzman-Muniz J. Moy-Lopez N. (2011). Stress by noise produces differential effects on the proliferation rate of radial astrocytes and survival of neuroblasts in the adult subgranular zone. Neurosci. Res. 70, 243–250 10.1016/j.neures.2011.03.013 21514330

[B19] Gould E. Mcewen B. S. Tanapat P. Galea L. A. Fuchs E. (1997). Neurogenesis in the dentate gyrus of the adult tree shrew is regulated by psychosocial stress and NMDA receptor activation. J. Neurosci. 17, 2492–2498 9065509 10.1523/JNEUROSCI.17-07-02492.1997 PMC6573503

[B20] Gu Y. Arruda-Carvalho M. Wang J. Janoschka S. R. Josselyn S. A. Frankland P. W. (2012). Optical controlling reveals time-dependent roles for adult-born dentate granule cells. Nat. Neurosci. 15, 1700–1706 10.1038/nn.3260 23143513 PMC3509272

[B21] Huang L. Bittman E. L. (2002). Olfactory bulb cells generated in adult male golden hamsters are specifically activated by exposure to estrous females. Horm. Behav. 41, 343–350 10.1006/hbeh.2002.1767 11971669

[B22] Kee N. Teixeira C. M. Wang A. H. Frankland P. W. (2007). Preferential incorporation of adult-generated granule cells into spatial memory networks in the dentate gyrus. Nat. Neurosci. 10, 355–362 10.1038/nn1847 17277773

[B23] Keller M. Meurisse M. Levy F. (2005). Mapping of brain networks involved in consolidation of lamb recognition memory. Neuroscience 133, 359–369 10.1016/j.neuroscience.2005.02.027 15885919

[B24] Keller M. Perrin G. Meurisse M. Ferreira G. Levy F. (2004). Cortical and medial amygdala are both involved in the formation of olfactory offspring memory in sheep. Eur. J. Neurosci. 20, 3433–3441 10.1111/j.1460-9568.2004.03812.x 15610176

[B25] Kendrick K. M. Lévy F. Keverne E. B. (1991). Importance of vaginocervical stimulation for the formation of maternal bonding in primiparous and multiparous parturient ewes. Physiol. Behav. 50, 595–600 10.1016/0031-9384(91)90551-x 1801015

[B26] Keverne E. B. Lévy F. Poindron P. Lindsay D. R. (1983). Vaginal stimulation: an important determinant of maternal bonding in sheep. Science 219, 81–83 10.1126/science.6849123 6849123

[B27] Keyser-Marcus L. Stafisso-Sandoz G. Gerecke K. Jasnow A. Nightingale L. Lambert K. G. (2001). Alterations of medial preoptic area neurons following pregnancy and pregnancy-like steroidal treatment in the rat. Brain Res. Bull. 55, 737–745 10.1016/s0361-9230(01)00554-8 11595357

[B28] Kinsley C. H. Madonia L. Gifford G. W. Tureski K. Griffin G. R. Lowry C. (1999). Motherhood improves learning and memory. Nature 402, 137–138 10647003 10.1038/45957

[B29] Kinsley C. H. Trainer R. Stafisso-Sandoz G. Quadros P. Marcus L. K. Hearon C. (2006). Motherhood and the hormones of pregnancy modify concentrations of hippocampal neuronal dendritic spines. Horm. Behav. 49, 131–142 10.1016/j.yhbeh.2005.09.001 16005000

[B30] Kopel H. Schechtman E. Groysman M. Mizrahi A. (2012). Enhanced synaptic integration of adult-born neurons in the olfactory bulb of lactating mothers. J. Neurosci. 32, 7519–7527 10.1523/jneurosci.6354-11.2012 22649230 PMC6703584

[B31] Larsen C. M. Grattan D. R. (2010). Prolactin-induced mitogenesis in the subventricular zone of the maternal brain during early pregnancy is essential for normal postpartum behavioral responses in the mother. Endocrinology 151, 3805–3814 10.1210/en.2009-1385 20484459

[B32] Lazarini F. Lledo P.-M. (2011). Is adult neurogenesis essential for olfaction? Trends Neurosci. 34, 20–30 10.1016/j.tins.2010.09.006 20980064

[B33] Lazarini F. Mouthon M. A. Gheusi G. De Chaumont F. Olivo-Marin J. C. Lamarque S. (2009). Cellular and behavioral effects of cranial irradiation of the subventricular zone in adult mice. PLoS One 4:e7017 10.1371/journal.pone.0007017 19753118 PMC2737283

[B34] Lehmann K. Butz M. Teuchert-Noodt G. (2005). Offer and demand: proliferation and survival of neurons in the dentate gyrus. Eur. J. Neurosci. 21, 3205–3216 10.1111/j.1460-9568.2005.04156.x 16026459

[B35] Lemaire V. Tronel S. Montaron M. F. Fabre A. Dugast E. Abrous D. N. (2012). Long-lasting plasticity of hippocampal adult-born neurons. J. Neurosci. 32, 3101–3108 10.1523/jneurosci.4731-11.2012 22378883 PMC6622037

[B36] Leuner B. Mirescu C. Noiman L. Gould E. (2007). Maternal experience inhibits the production of immature neurons in the hippocampus during the postpartum period through elevations in adrenal steroids. Hippocampus 17, 434–442 10.1002/hipo.20278 17397044

[B37] Lévy F. Keller M. (2008). “Neurobiology of maternal behavior in sheep,” in Advances in the Study of Behavior, eds Brockmann H. J. Roper T. J. Naguib M. Wynne-Edwards K. E. Barnard C. Mitani J. C. (San Diego, California, USA: Elsevier Inc., Academic Press), 399–437

[B38] Lévy F. Keller M. (2009). Olfactory mediation of maternal behavior in selected mammalian species. Behav. Brain. Res. 200, 336–345 10.1016/j.bbr.2008.12.017 19146885

[B39] Lévy F. Gheusi G. Keller M. (2011). Plasticity of the parental brain: a case for neurogenesis. J. Neuroendocrinol. 23, 984–993 10.1111/j.1365-2826.2011.02203.x 21824205

[B40] Lévy F. Guevara-Guzman R. Hinton M. R. Kendrick K. M. Keverne E. B. (1993). Effects of parturition and maternal experience on noradrenaline and acetylcholine release in the olfactory bulb of sheep. Behav. Neurosci. 107, 662–668 10.1037/0735-7044.107.4.662 8397870

[B41] Lévy F. Keller M. Poindron P. (2004). Olfactory regulation of maternal behavior in mammals. Horm. Behav. 46, 284–302 10.1016/j.yhbeh.2004.02.005 15325229

[B42] Lévy F. Keller M. Cornilleau F. Moussu C. Ferreira G. (2010). Vaginocervical stimulation of ewes induces the rapid formation of a new bond with an alien young without interfering with a previous bond. Dev. Psychobiol. 52, 537–544 10.1002/dev.20459 20806326

[B43] Lévy F. Locatelli A. Piketty V. Tillet Y. Poindron P. (1995). Involvement of the main but not the accessory olfactory system in maternal behavior of primiparous and multiparous ewes. Physiol. Behav. 57, 97–104 10.1016/0031-9384(94)00200-o 7878131

[B44] Lévy F. Poindron P. Le Neindre P. (1983). Attraction and repulsion by amniotic fluids and their olfactory control in the ewe around parturition. Physiol. Behav. 31, 687–692 10.1016/s0031-9384(83)80004-3 6665057

[B45] Livneh Y. Mizrahi A. (2011). Experience-dependent plasticity of mature adult-born neurons. Nat. Neurosci. 15, 26–28 10.1038/nn.2980 22081159

[B46] Lu L. Bao G. Chen H. Xia P. Fan X. Zhang J. (2003). Modification of hippocampal neurogenesis and neuroplasticity by social environments. Exp. Neurol. 183, 600–609 10.1016/s0014-4886(03)00248-6 14552901

[B47] Lussier A. L. Lebedeva K. Fenton E. Y. Guskjolen A. Caruncho H. J. Kalynchuk L. E. (2013). The progressive development of depression-like behavior in corticosterone-treated rats is paralleled by slowed granule cell maturation and decreased reelin expression in the adult dentate gyrus. Neuropharmacology 71, 174–183 10.1016/j.neuropharm.2013.04.012 23608736

[B48] Mak G. K. Enwere E. K. Gregg C. Pakarainen T. Poutanen M. Huhtaniemi I. (2007). Male pheromone-stimulated neurogenesis in the adult female brain: possible role in mating behavior. Nat. Neurosci. 10, 1003–1011 10.1038/nn1928 17603480

[B49] Mandairon N. Sacquet J. Garcia S. Ravel N. Jourdan F. Didier A. (2006). Neurogenic correlates of an olfactory discrimination task in the adult olfactory bulb. Eur. J. Neurosci. 24, 3578–3588 10.1111/j.1460-9568.2006.05235.x 17229106

[B50] Ming G. L. Song H. (2005). Adult neurogenesis in the mammalian central nervous system. Annu. Rev. Neurosci. 28, 223–250 10.1146/annurev.neuro.28.051804.101459 16022595

[B51] Mitra R. Sundlass K. Parker K. J. Schatzberg A. F. Lyons D. M. (2006). Social stress-related behavior affects hippocampal cell proliferation in mice. Physiol. Behav. 89, 123–127 10.1016/j.physbeh.2006.05.047 16837015

[B52] Moreno M. M. Linster C. Escanilla O. Sacquet J. Didier A. Mandairon N. (2009). Olfactory perceptual learning requires adult neurogenesis. Proc. Natl. Acad. Sci. U S A 106, 17980–17985 10.1073/pnas.0907063106 19815505 PMC2764902

[B53] Mouret A. L. Gheusi G. Gabellec M.-M. De Chaumont F. Olivo-Marin J.-C. Lledo P.-M. (2008). Learning and survival of newly generated neurons: when time matters. J. Neurosci. 28, 11511–11516 10.1523/jneurosci.2954-08.2008 18987187 PMC6671302

[B54] Munaro N. I. (1990). Maternal behavior: glutamic acid decarboxylase activity in the olfactory bulb of the rat. Pharmacol. Biochem. Behav. 36, 81–84 10.1016/0091-3057(90)90129-6 2349273

[B55] Oboti L. Schellino R. Giachino C. Chamero P. Pyrski M. Leinders-Zufall T. (2011). Newborn interneurons in the accessory olfactory bulb promote mate recognition in female mice. Front. Neurosci. 5:113 10.3389/fnins.2011.00113 21994486 PMC3182443

[B56] Ormerod B. Galea L. (2001). Reproductive status influences cell proliferation and cell survival in the dentate gyrus of adult female meadow voles: a possible regulatory role for estradiol. Neuroscience 102, 369–379 10.1016/s0306-4522(00)00474-7 11166123

[B57] Pawluski J. L. Galea L. A. (2007). Reproductive experience alters hippocampal neurogenesis during the postpartum period in the dam. Neuroscience 149, 53–67 10.1016/j.neuroscience.2007.07.031 17869008

[B58] Rochefort C. Lledo P. M. (2005). Short-term survival of newborn neurons in the adult olfactory bulb after exposure to a complex odor environment. Eur. J. Neurosci. 22, 2863–2870 10.1111/j.1460-9568.2005.04486.x 16324121

[B59] Ruscio M. G. Sweeny T. D. Hazelton J. L. Suppatkul P. Boothe E. Carter C. S. (2008). Pup exposure elicits hippocampal cell proliferation in the prairie vole. Behav. Brain. Res. 187, 9–16 10.1016/j.bbr.2007.08.028 17913255 PMC2699755

[B60] Sahay A. Wilson D. A. Hen R. (2011). Pattern separation: a common function for new neurons in hippocampus and olfactory bulb. Neuron 70, 582–588 10.1016/j.neuron.2011.05.012 21609817 PMC3109085

[B61] Sakamoto M. Imayoshi I. Ohtsuka T. Yamaguchi M. Mori K. Kageyama R. (2011). Continuous neurogenesis in the adult forebrain is required for innate olfactory responses. Proc. Natl. Acad. Sci. U S A 108, 8479–8484 10.1073/pnas.1018782108 21536899 PMC3100923

[B62] Sanchez-Andrade G. James B. M. Kendrick K. M. (2005). Neural encoding of olfactory recognition memory. J. Reprod. Dev. 51, 547–558 10.1262/jrd.17031 16284449

[B63] Shingo T. Gregg C. Enwere E. Fujikawa H. Hassam R. Geary C. (2003). Pregnancy-stimulated neurogenesis in the adult female forebrain mediated by prolactin. Science 299, 117–120 10.1126/science.1076647 12511652

[B64] Siegel S. (1956). Nonparametric Statistics for the Behavioral Sciences. Tokyo: McGraw-Hill

[B65] Stern J. E. Armstrong W. E. (1998). Reorganization of the dendritic trees of oxytocin and vasopressin neurons of the rat supraoptic nucleus during lactation. J. Neurosci. 18, 841–853 9437006 10.1523/JNEUROSCI.18-03-00841.1998 PMC6792760

[B66] Sultan S. Mandairon N. Kermen F. Garcia S. Sacquet J. Didier A. (2010). Learning-dependent neurogenesis in the olfactory bulb determines long-term olfactory memory. FASEB J. 24, 2355–2363 10.1096/fj.09-151456 20215526

[B67] Tronel S. Fabre A. Charrier V. Oliet S. H. Gage F. H. Abrous D. N. (2010). Spatial learning sculpts the dendritic arbor of adult-born hippocampal neurons. Proc. Natl. Acad. Sci. U S A 107, 7963–7968 10.1073/pnas.0914613107 20375283 PMC2867872

[B68] Valley M. T. Mullen T. R. Schultz L. C. Sagdullaev B. T. Firestein S. (2009). Ablation of mouse adult neurogenesis alters olfactory bulb structure and olfactory fear conditioning. Front. Neurosci. 3:51 10.3389/neuro.22.003.2009 20582278 PMC2858604

[B69] Wong E. Y. Herbert J. (2004). The corticoid environment: a determining factor for neural progenitors’ survival in the adult hippocampus. Eur. J. Neurosci. 20, 2491–2498 10.1111/j.1460-9568.2004.03717.x 15548194 PMC1592224

[B70] Wong E. Y. Herbert J. (2006). Raised circulating corticosterone inhibits neuronal differentiation of progenitor cells in the adult hippocampus. Neuroscience 137, 83–92 10.1016/j.neuroscience.2005.08.073 16289354 PMC2651634

